# The impact of Brazil’s transport network on the spread of COVID-19

**DOI:** 10.1038/s41598-022-27139-1

**Published:** 2023-02-08

**Authors:** Giovanna Cavali Silva, Evandro Marcos Saidel Ribeiro

**Affiliations:** 1 PECE Programa de Educação Continuada, Escola Politécnica da Universidade de São Paulo, Sao Paulo, 05508-030 Brazil; 2 Faculdade de Economia Administração e Contabilidade de Ribeirão Preto, Universidade de São Paulo, Ribeirão Preto, 14040-905 Brazil

**Keywords:** Infectious diseases, Computer science

## Abstract

The transport network between cities is key in understanding epidemic outbreaks, especially in a vast country like Brazil with 5569 cities spread out over 8.5 million square kilometers. In order to study the COVID-19 spread in Brazil, we built a transport network where each city is a node and the edges are connections by land and air. Our findings have shown that by adding air connections, the average path length substantially decreases (70%) while the clustering coefficient remains almost unchanged, very typical of small-world networks. The airways are shortcuts connecting previously distant cities and hubs, therefore shrinking the distances in the network. Also, the cities with airports are central nodes, which makes them dissemination hotspots and key targets for interventions.

## Introduction

### COVID-19 pandemic

Reports of a potentially lethal and pandemic new viral infection surfaced in Wuhan Province, China, in late 2019. It was clear from early reports that this new disease, later known as COVID-19, had severe respiratory complications and could have an alarming lethality rate, in addition to its high transmission rate. And despite several measures, the virus has spread and the world has experienced a widespread pandemic. COVID-19 infections require a physical mechanism of transmission between infected and susceptible individuals. This does not have to be physical contact, but it can arise when individuals are close enough and together long enough for a pathogen to pass from one individual to another^1^. Therefore, the connections between individuals (or group of individuals) that allow an infectious disease, such as COVID-19, to propagate can be described as a network, where nodes are individuals and edges are infectious attempts or transmission events or simply individuals who are in contact^2^. And then, starting with several infected nodes, the epidemic proceeds by transmission over the edges.

While the interactions in a population can define a network, the network that is generated can provide insights into the epidemiological dynamics and be part of disease control. For example, contact network analysis can improve predictions of the likely distribution of an infection, and also identify probable transmission network connections and hence treat or contain their contacts thereby reducing the spread of infection^3^. Using the same idea, several researchers have used network science to understand COVID-19 transmission dynamics and the effects of controls strategies^4–8^.

### Network applications in epidemiology

Susceptible-Infected-Recovered (SIR) model, proposed by Kermack et al.^9^, was one of the first epidemiological models and considered the standard model of epidemic spread. In the SIR model, society is divided into three states: susceptible (S) which contains individuals who are vulnerable and not yet infected; infected (I) which is formed from susceptible individuals who become infected, and in this state, they can spread the disease; recovered (R) that consists of previously infected individuals who have overcome the disease and have acquired some level of immunity (if recovery does not result in long-term immunity, a Susceptible-Infected-Susceptible (SIS) model may be appropriate). The experience with the SIR model has motivated many variations and many of these models incorporate additional states to account for exposed (E) where individuals are infected but not yet infectious, vaccinated (V), and quarantined (Q).

When applying the SIR model and its variation to an existing virus or epidemic, the states and the contact network are selected based on the characteristics of a specific disease and the purpose of the model. The contact network has been changing drastically due to the expansion of air-sea-land transportation in range, travel speed, and volume of passengers and goods transported, taking with them pathogens and their vectors. According to Tatem et al.^10^, three important consequences of the expansion of the global transport network are infectious disease pandemics, vector invasion events, and the import of vector-borne pathogens.

Browne et al.^11^ studied how air, land, and maritime transport hubs are associated with the spread of influenza and coronaviruses. And they concluded that air transport accelerates and amplifies the spread of epidemics, where the transmission can occur on board planes, at destinations, and at airports. In a transportation network, the distances can be misleading when dealing with different modes of transportation. In that sense, Brockmann et al.^12^ replaced conventional geographic distance with probabilistically motivated effective distance in order to effectively predict the behavior of an air-traffic-mediated epidemic.

In order to describe the early COVID-19 global country-to-country transmission, Hancean et al.^13^ studied the impact of human mobility networks on COVID-19 onset in 203 different countries and found that migration and tourism inflow increases the probability of COVID-19 case importations. Also, air flights were the dominant mode of transportation and returning travelers were the main carriers.

Based on mathematical models, Nicolelis et al.^14^ analyzed the factors that influenced the dynamics of COVID-19 virus propagation in Brazil and concluded that in the first 3 months of the pandemic, the city of São Paulo (SP) was responsible for more than 85% of case propagation, and this number reaches 98-99% if we add 16 other capitals. In addition, 26 federal highways were responsible for 30% of the spread of cases.^15^ also proved the importance of highways in the spread of COVID-19 in Brazil.

To simulate the spread of epidemics using networks, Kuzdeuov et al.^7^ model the country as a network and each city as a node. Each node runs an SEIR (Susceptible-Exposed-Infected-Recovered) model and the population transfer between nodes is considered using transport networks that allow modeling the geographic spread of the disease. The simulator was calibrated with real data from the COVID-19 pandemic in Lombardy, Italy. Then, the epidemic situation in Kazakhstan as of 31 May 2020 was accurately recreated using the model.

Using the same idea in Brazil’s landscape, we have studied Brazil’s contact network in order to identify important super-spreader nodes and edges that are epidemiological hotspots for the spread of the pandemic and also studied the role of transportation in the dissemination of the disease.

## Methodology

### Transport network

To build the network for the study, we first focused on the connections by land. From the municipal grid database publicly available on the IBGE (Instituto Brasileiro de Geografia e Estatísticas) website^16^, and using the python library *geopandas* and *networkx*, we were able to build the road network (G1), using the following assumptions: each city consists of a node located in the centroid of the city area, and the cities that were physically neighboring each other were connected by an edge. This way we were able to simplify Brazil’s highway grid, reducing the number of edges and facilitating the network calculations, but keeping the most important connections.

Then, using the database of national flights from 2018 to 2020 from ANAC (Agência Nacional de Aviação Civil)^17^, edges were added between all the cities pairs that had air travel routes connecting them in the period analyzed, resulting in G2 network.

### Simulation

To understand the disease dynamic in the networks, we simulated the infection spreading based on the work of Yager and Taylor^18^, using the assumptions below:

- The states for the simulation are susceptible (S) and infected (I). Since we are focusing on the beginning of the pandemic in Brazil, it doesn’t make a lot of sense to talk about cities recovered. Thus in the analysis, the cities will be either susceptible or infected. And a city is considered infected when it has at least one case confirmed.
- The probability of infection was fixed at 25%. This was calibrated empirically using Brazil’s COVID-19 cases dataset publicly available in Brasil.io repository^19^ for the first 40 epidemiological weeks, where each epidemiological week is equivalent to an iteration in our model. The dataset includes daily information on COVID-19 cases and deaths (new and overall) for every city in Brazil since March 2020, when the first case was confirmed in the country.
- At every iteration, all the susceptible neighbors of an infected city have a 25% chance to be infected and start infecting others in the next iteration. And neighbors are all cities connected by land or air.
- The city of Sao Paulo (SP) started the simulation infected.

### Network metrics

Using the library *networkx* we calculated network metrics and algorithms to extract insights from Brazil’s transport network. The network metrics used were: the number of nodes and edges; the maximum, minimum, and average degree (degree of a node is the number of neighbors, nodes adjacent to it); the average clustering coefficient (clustering coefficient is the probability that two neighbors of a given node are themselves, neighbors; in other words, the probability of three nodes forming a triangle in the network); average path length, which is is the average of the shortest paths between all pairs of nodes from a network; and diameter of a network which is the longest shortest path.

Also studied centrality of the network, which is a way to quantify the importance of a node or edge. The centrality importance can have different definitions such as the amount of degree (degree centrality), average proximity to other nodes (closeness centrality), the fraction of shortest paths that pass through a node (betweeness centrality), etc. The centralities were also calculated using library *networkx*.

And finally, used Kruskal’s algorithm^20^, implemented by library *networkx*, to calculate the minimum spanning tree. A spanning tree is a subset of a graph, which has all the vertices with the minimum possible number of edges. Hence, a spanning tree does not have cycles and it cannot be disconnected. It is basically used to find a minimum path to connect all nodes in a graph. In a weighted graph, a minimum spanning tree is a spanning tree that has a minimum weight compared to all other spanning trees of the same graph. In real-world situations, this weight can be measured as distance, congestion, traffic load, or any arbitrary value denoted to the edges. Here, we used the algorithm to find the most important paths connecting the cities and possibly find the ones responsible for the widespread pandemic in Brazil. Since the distances can be misleading when comparing different modes of transportation^12^, the weight for the minimum spanning tree algorithm was calculated as the inverse of the euclidean distance between nodes (city’s centroids). That’s how we managed to prioritize the airways since that’s where most of the transmission occurred.

## Results

### Simulation

**Figure 1:**
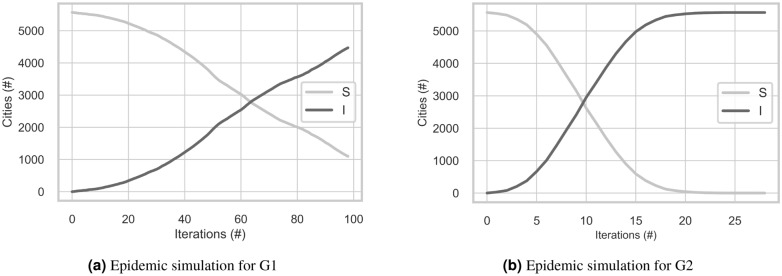
Epidemic simulation.

**Figure 2:**
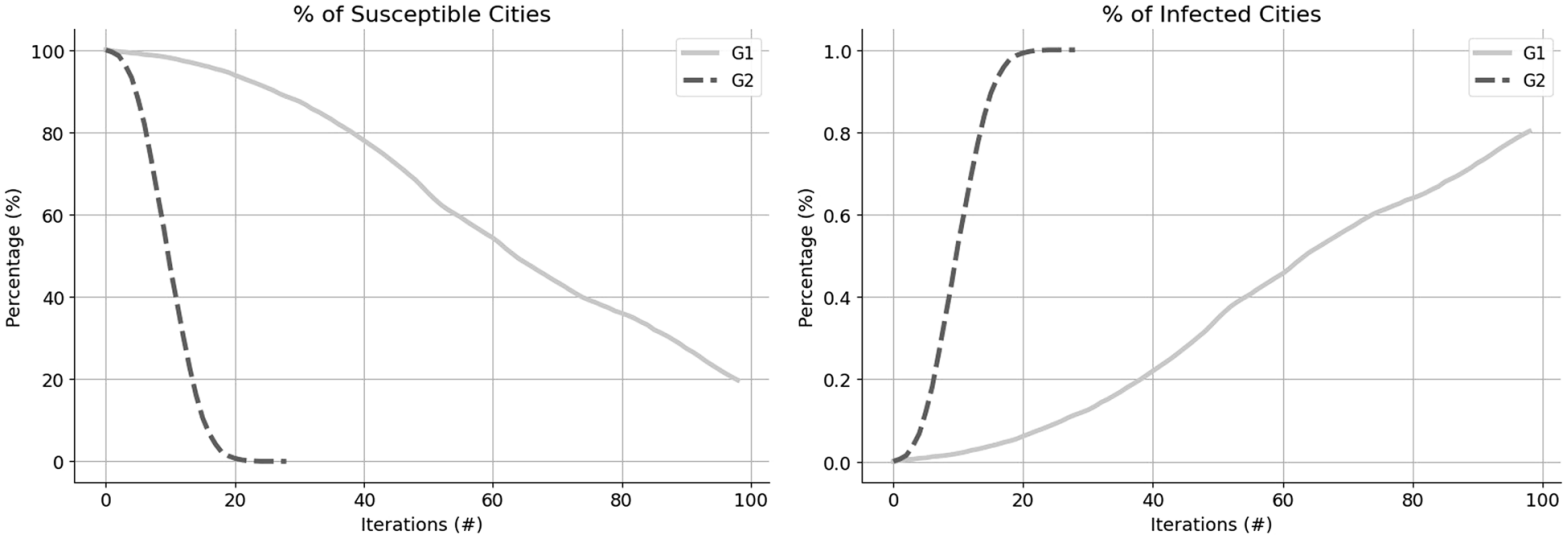
Epidemic simulations comparison.

We performed an epidemic simulation in both networks and the results are shown in Fig. 1. Even though both graphs look similar, please be aware of the x-axis: for G1 the simulation stops at iteration 100th, and for G2, at 28th. We encountered completely different scenarios as you can see more clearly in Fig. 2 where the percentage of Susceptible and Infected nodes were plotted. Both simulations had the same starting point, and after 28 iterations, all the 5569 nodes from G2 had already been infected and there was not one susceptible node left, meanwhile, only 12% of the cities in G1 had experienced any contact with the disease. This result shows how the airways can changes the dynamic of disease transmission in Brazil: while in G2, in only a few iterations, the disease had already spread to all the regions, in G1, the pace is very slow and in 28 iterations, the disease was still very much contained to the region where it started.

The main goal of the simulation was to show the change in transmission when airways are added to the network. Using the simulation to predict cases is out of the scope of this study, therefore we are using a simplified version of SIR’s model.

### Network metrics and parameters

The calculated metrics are shown in Table 1. As expected, the number of edges is greater for graph G2 if we compare it to G1. And we can also see a difference in the number of nodes because there is a relevant island that does not neighbor any other city by land.

**Table 1:** Network metrics.

Metric	G1	G2
Number of nodes	5568	5569$^{\textrm{a}}$
Number of edges	16,470	17,437
Maximum degree	23	95
Minimum degree	1	1
Average degree	5.9160	6.2622
Median degree	6.0	6.0
Average distance	27.2563	7.2337
Diameter	78	16
Avg clustering coefficient	0.4801	0.4787

$^{\textrm{a}}$ Fernando de Noronha was added to the network.

Intuitively, the higher the degree of the node, the higher the number of neighbors and the more likely it is to be neighbor of an already infected node. Also, the more neighbors a node has, the more likely it is to cause a large number of onward cases. Even though G2 has more edges, both networks have comparable average degree. This is because the new edges have little influence in the number of neighbors overall since its impact is restricted to a small portion of the cities that have airports.

G1 and G2 have similar clustering coefficients, and therefore, we would expect that the spread of the pandemic would have a similar pace in both cases. But the SIR Simulation says otherwise and this is due to the average path length. The airways shrunk the average distance between the cities from 27.2563 to 7.2337, as well as the diameter of the network.

Two properties of many real-world networks are that the distance between any pairs of nodes is relatively small while at the same time the clustering is relatively high. Watts^21^ explains how the clustering coefficient and path length evolves with alpha, which is a parameter that describes how random is the probability that two nodes are connected. It starts with a “caveman world”, where a node is most likely to connect to a neighbor of its neighbor, and clustering coefficient and average path length are high and alpha, small. By increasing alpha, we get to a point where the path length drops substantially while the clustering coefficient remains high, and that’s where we see the small world property, where a vast majority of nodes are reachable in a small number of steps. This notion has been popularized by terms like the “six degrees of separation” between any two people, meaning they are typically connected by a chain of six or fewer edges in a social network graph. And we can see the same behavior in Brazil’s cities network G2 where it only takes a short number of steps to reach every city and diseases are able to spread much more rapidly.

**Figure 3:**
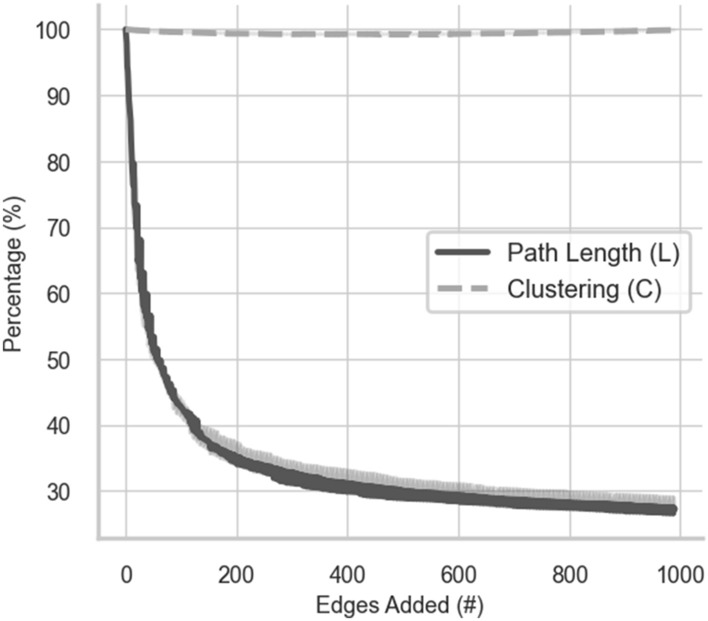
Transition from G1 to G2: clustering coefficient and average path length.

To understand the transition between G1 and G2, we randomly added the air connections to G1 one by one and calculated the clustering coefficient and the average path length of the new network. And repeated the process until we reached G2. The analysis resulted in the graph below (Fig. 3), where we can see that, by adding 967 edges, the clustering coefficient remains almost unchanged, while the path length decrease by 70%. On one side, we have a very clustered network where the nodes are regionally connected to one another similar to a lattice. And once we add the airways, shortcuts are created contracting the path length between previously distant nodes.

### Network centrality

Central nodes are likely to become infected early on in the epidemic and are also key targets for intervention^3^, so finding the central nodes is crucial when trying to minimize its impact.

**Figure 4:**
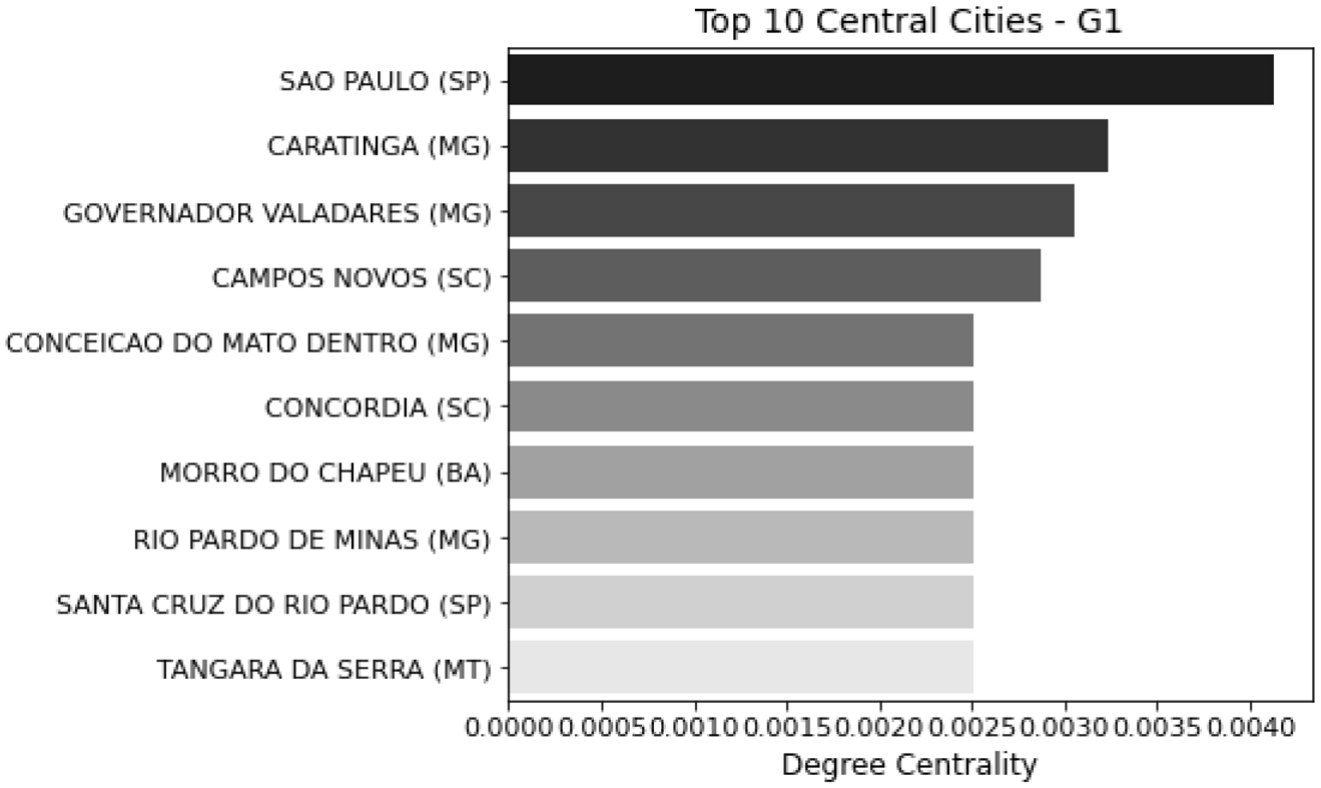
Top 10 central cities for G1.

**Figure 5:**
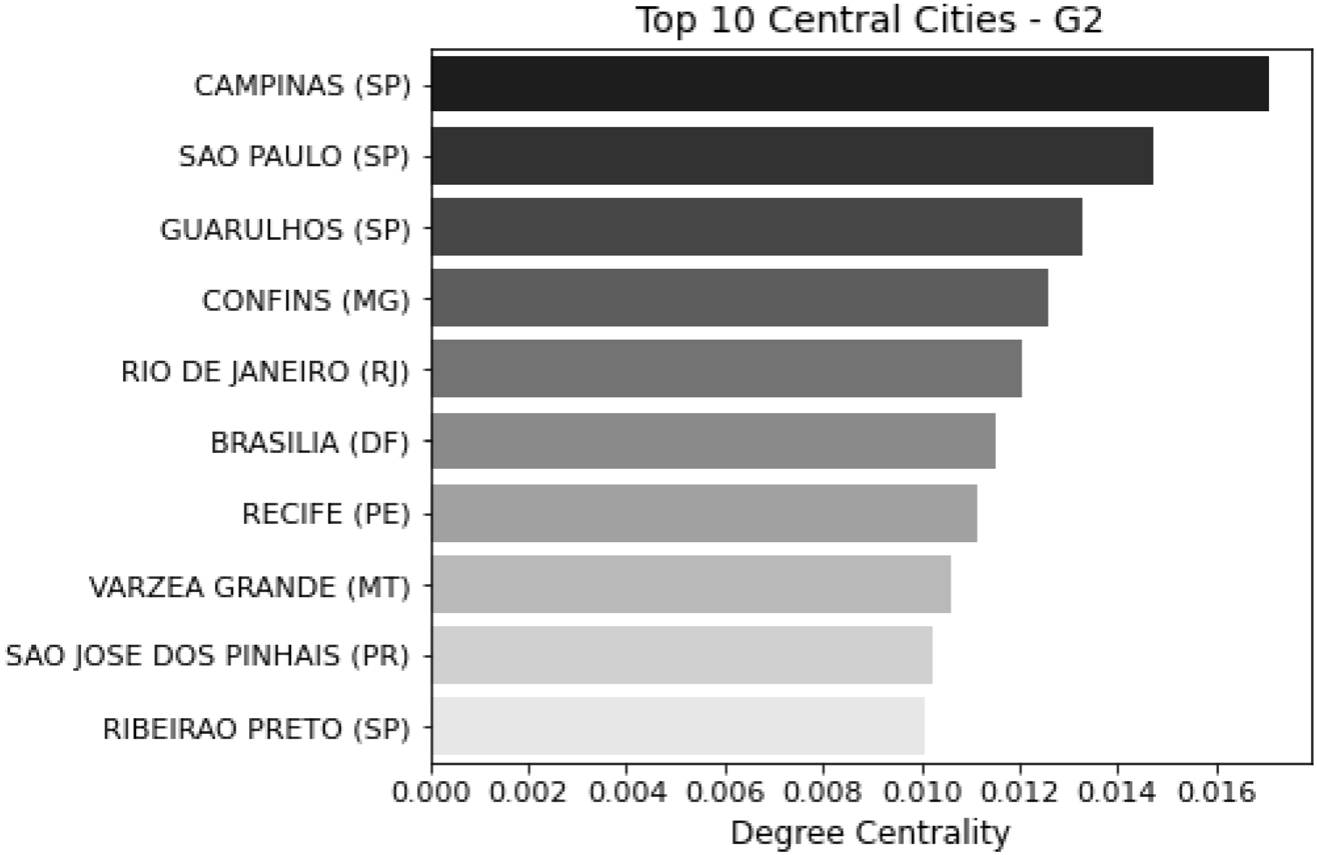
Top 10 central cities for G2.

For all the centralities studied (degree centrality, closeness centrality and betweeness centrality), the most important nodes in G2 are the cities with the largest airports and intense flow of people such as Campinas (SP), São Paulo (SP), Guarulhos (SP), Confins (MG) and Rio de Janeiro (RJ). On the other hand, the top centrality cities in G1 are cities located in the center of Brazil that geographically neighbor a large number of cities but are not necessarily important transmission hubs. Figures 4 and 5 show the top 10 central cities in G1 and G2, ranked by the degree centrality algorithm.

### Minimum spanning tree

**Figure 6:**
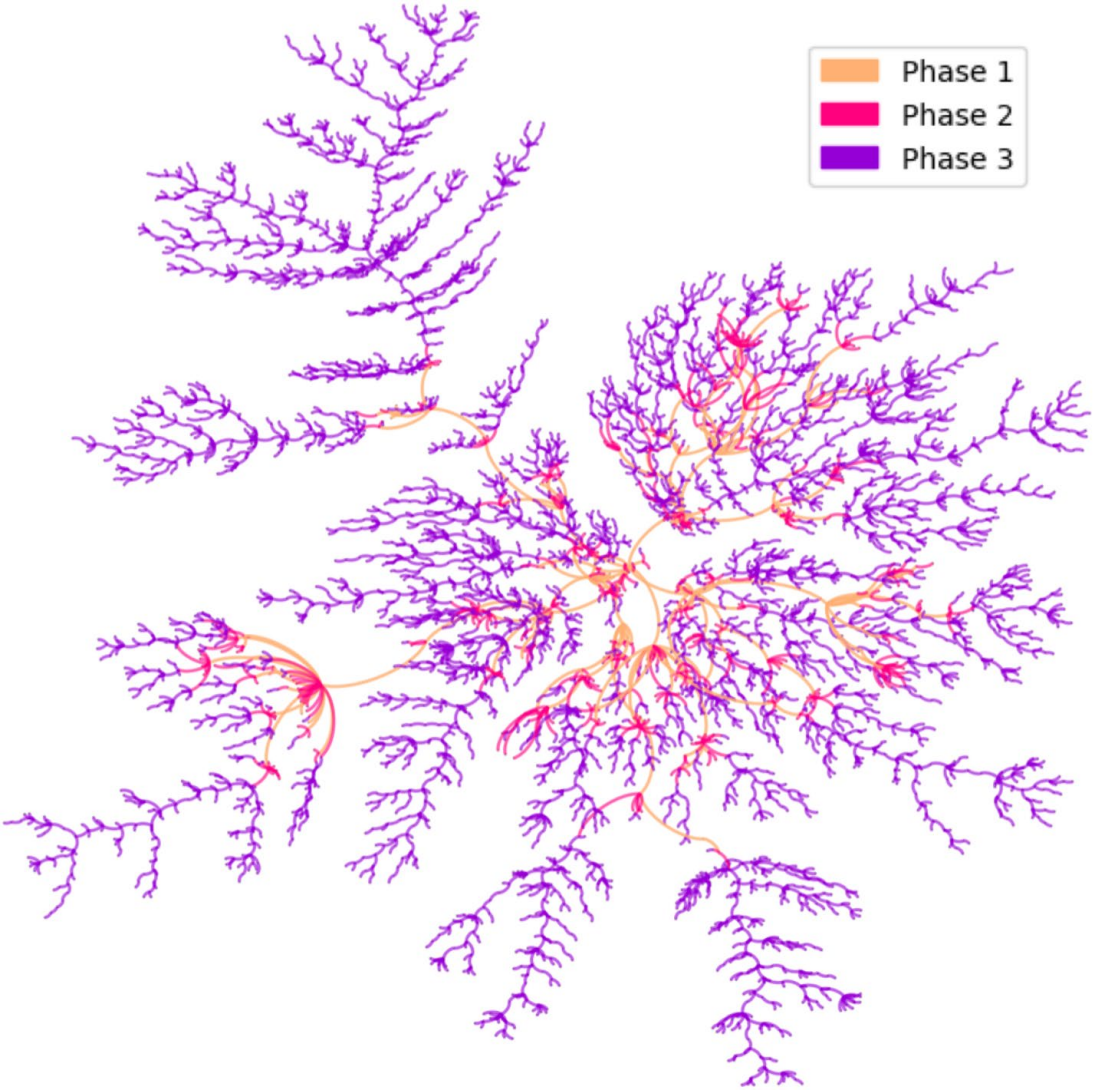
Minimum spanning tree: stages of COVID-19 contamination.

In order to obtain the most important transmission paths, Kruskal’s minimum spanning tree algorithm^20^, implemented in python using library *networkx*, was applied to G2. As expected, the airways in the minimum spanning tree are connecting distant regions that are connected locally to other cities by land, regions that would be isolated without the airports. From the minimum spanning tree, we can draw an analogy with the COVID-19 dynamics in Brazil, as you can see in Fig. 6. The virus entered the country first in March of 2020 at all major Brazilian international airports (Phase 1) and quickly spread to other local airports and its metropolitan area (Phase 2). From that point on, the small group of these large cities began spreading COVID-19 throughout the entire country, through the extensive highway grid (Phase 3).

## Conclusion

Our findings have confirmed that the airways have shrunken substantially the distances between cities in Brazil, illustrated by a 70% decline in the average path length between nodes. A low average path length between cities indicates greater integration among geographical regions and the ease of information flow, but also, when we are talking about epidemics, the ease of virus spreading. This fact is demonstrated in the simulation done in “Simulation”. The COVID-19 cases dataset^19^ shows that 99% of the cities of Brazil had already been infected in the 20th epidemiological week, and that is much more close to what happened in G2 simulation if compared to G1.

As expected, the most important cities, the ones with the highest centrality, were the cities with major airports. Those cities are very important for transportation since they connect different parts of the country, but at the same time, they were the gateway for the first COVID-19 cases and responsible for the dissemination of the virus to different regions. Those cities had a very important role in the early stages of the COVID-19 epidemic in Brazil and should have been the target of severe intervention before the first case was even confirmed. Once the virus arrived in medium-sized cities with local airports, it then spread to smaller cities and towns through the extensive highway grid. And in that stage, the transmission hubs have become numerous and the strategies to contain the outbreak should be done at a country level. We were able to emulate the virus dynamic using a minimum spanning tree in “Minimum spanning tree”.

From this study, we can see how a small world transport network is fragile when it comes to epidemics: once the virus has entered the network, the impacts are overwhelming in a short period of time. And the real transport network does not have only roads connecting neighboring cities and flights, there are highways connecting distant cities and also maritime and rail transportation that creates more shortcuts therefore the real distance between cities is expected to be smaller than the one calculated by our model. And the impact of a highly infectious disease like COVID-19 can be devastating unless there are severe and generalized interventions.

## Supplementary Information

Supplementary Information 1.

Supplementary Information 2.

Supplementary Information 3.

## Data Availability

Brazil’s COVID-19 cases dataset analyzed during the current study is publicly available in the Brasil.io repository, https://brasil.io/dataset/covid19/caso/. Brazil’s municipal grid database is publicly available in the IBGE (Instituto Brasileiro de Geografia e Estatísticas) repository, https://www.ibge.gov.br/geociencias/organizacao-do-territorio/15774-malhas.html?= &t=downloads. Brazil’s national flights database is publicly available in the ANAC (Agência Nacional de Aviação Civil) repository, https://www.gov.br/anac/pt-br/assuntos/dados-e-estatisticas. The publicly available datasets used in the study are in the Supplementary Information. The datasets generated during the current study are available from the corresponding author on reasonable request.

## References

[1] Bell J (2021). Beyond COVID-19: Network science and sustainable exit strategies. J. Phys. Complex..

[2] Pellis L (2015). Eight challenges for network epidemic models. Epidemics.

[3] Danon L (2011). Networks and the epidemiology of infectious disease. Interdiscip. Perspect. Infect. Dis..

[4] Firth J (2020). Using a real-world network to model localized covid-19 control strategies. Nat. Med..

[5] Zhu S, Kou M, Lai F, Feng Q, Du G (2021). The connectedness of the coronavirus disease pandemic in the world: A study based on complex network analysis. Front. Phys..

[6] Thurner S, Klimek P, Hanel R (2020). A network-based explanation of why most covid-19 infection curves are linear. Proc. Natl. Acad. Sci..

[7] Askat K (2020). A network-based stochastic epidemic simulator: Controlling covid-19 with region-specific policies. IEEE J. Biomed. Health Inform..

[8] Uhlig S, Nichani K, Uhlig C, Simon K (2020). Modeling projections for covid-19 pandemic by combining epidemiological, statistical, and neural network approaches. medRxiv.

[9] Ogilvy KW, McKendrick AG (1927). A contribution to the mathematical theory of epidemics. Proc. R. Soc. Lond..

[10] Tatem, A., Rogers, D. & Hay, S. *Global Transport Networks and Infectious Disease Spread*, vol. 62 *of Advances in Parasitology* (Academic Press, 2006). 10.1016/S0065-308X(05)62009-X PMC3145127 16647974

[11] Browne A, St-Onge Ahmad S, Beck C, Nguyen-Van-Tam J (2015). The roles of transportation and transportation hubs in the propagation of influenza and coronaviruses: A systematic review. J. Travel Med..

[12] Brockmann D, Helbing D (2013). The hidden geometry of complex, network-driven contagion phenomena. Science.

[13] Hâncean M-G, Slavinec M, Perc M (2021). The impact of human mobility networks on the global spread of COVID-19. J. Complex Netw..

[14] Nicolelis MAL, Raimundo RLG, Peixoto PS, Andreazzi CS (2021). The impact of super-spreader cities, highways, and intensive care availability in the early stages of the covid-19 epidemic in brazil. Nature.

[15] Carmo RF, Nunes BEBR, Machado MF, Armstrong AC, Souza CDF (2020). Expansion of COVID-19 within Brazil: The importance of highways. J. Travel Med..

[16] IBGE. Malha de municícipios do brazil. https://www.ibge.gov.br/geociencias/organizacao-do-territorio/15774-malhas.html?= &t=downloads. Último acesso em 30/1/2021.

[17] ANAC. Base de dados estatísticos do transporte aéreo. https://www.anac.gov.br/assuntos/setor-regulado/empresas/envio-de-informacoes/base-de-dados-estatisticos-do-transporte-aereo. Último acesso em 30/1/2021.

[18] Yager, N. A. & Taylor, M. Edge-based control of disease propagation through the world-wide airport network. https://github.com/nicholasyager/airport-disease-modeling-edge-based-control-of-disease-propagation-through-the-world-wide-airport-network (2014).

[19] Brasil.io. Covid-19 casos. https://brasil.io/dataset/covid19/caso/. Último acesso em 30/1/2021.

[20] Kruskal JB (1956). On the shortest spanning subtree of a graph and the traveling salesman problem. Proc. Am. Math. Soc..

[21] Watts, D. J. *Six Degrees: The Science of a Connected Age* (W. W. Norton & Company, 2004).

